# FUNCTIONAL STATUS ASSOCIATED WITH RISK OF READMISSION DURING HOME HEALTH CARE FOR PATIENTS WITH DEMENTIA

**DOI:** 10.1093/geroni/igz038.3033

**Published:** 2019-11-08

**Authors:** Sara Knox, Brian Downer, Allen Haas, Addie Middleton, Kenneth Ottenbacher

**Affiliations:** 1 MGH Institute of Health Professions, Boston, Massachusetts, United States; 2 University of Texas Medical Branch, Galveston, TX, Galveston, Texas, United States; 4 Medical College of South Carolina, Charleston, South Carolina, United States

## Abstract

Approximately 14.0% of Medicare beneficiaries are readmitted to a hospital within 30-days of home health admission. Individuals with dementia account for 30% of all home health care admissions and are at high-risk for rehospitalizations. Our primary objective was to determine the association between functional status and social support at admission to home health and 30-day potentially preventable readmissions (PPR) during home health care. We conducted a retrospective cohort study of 124,119 Medicare beneficiaries receiving home health (7/2013 – 6/2015) and diagnosed with dementia (ICD-9 codes). Approximately 65% of participants were over the age of 81, 61% were female, and 80% were Caucasian. The primary outcome was 30-day PPR during home health. OASIS items were used to create mobility, self-care, social support, and cognition categories. The overall rate of 30-day PPR was 7.6% (95% CI: 7.4-7.7) but varied by patient and health care utilization characteristics. After adjusting for sociodemographic and clinical characteristics, the odds ratios (OR) for the most dependent score quartile versus the most independent was 1.68(1.56,1.80 95% CI) for mobility, 1.78 (95% CI: 1.66- 1.91) for self-care, and 1.10(95%CI: 1.03-1.17) for social support. The OR for impaired versus intact cognition was 1.12 (95% CI: 1.05-1.20). Impaired functional and cognitive status as well as limited social support at admission to home health care are associated with increased risk of PPR for individuals with dementia. Future research is needed to determine if strategies targeted at mobility and self-care can decrease PPR during home health for individuals with severe dementia.

